# Temporal Variation and Ecological Risk Assessment of Metals in Soil Nearby a Pb–Zn Mine in Southern China

**DOI:** 10.3390/ijerph15050940

**Published:** 2018-05-09

**Authors:** Congcong Cao, Li Wang, Hairong Li, Binggan Wei, Linsheng Yang

**Affiliations:** 1 Key Laboratory of Land Surface Pattern and Simulation, Institute of Geographic Sciences and Natural Resources Research, Chinese Academy of Sciences, 11 A Datun Road, Beijing 100101, China; caocc.14b@igsnrr.ac.cn (C.C.); wangli@igsnrr.ac.cn (L.W.); lihr@igsnrr.ac.cn (H.L.); 2 College of Resources and Environment, University of Chinese Academy of Sciences, Beijing 100049, China

**Keywords:** tailing, metals, ecological risk, metal profile, metal distribution

## Abstract

Metal contamination in soil from tailings induces risks for the ecosystem and for humans. In this study, the concentrations and ecological risks of Cd, Cu, Pb, and Zn in soil contaminated by a tailing from Yangshuo (YS) lead and zinc (Pb–Zn) mine, which collapsed for more than 40 years, were determined in 2015. The mean concentrations of Zn, Pb, Cu, and Cd were 1301.79, 768.41, 82.60, and 4.82 mg/kg, respectively, which, with years of remediation activities, decreased by 66.9%, 61.7%, 65.4%, and 65.3% since 1986, but still exceed the national standards. From 1986 to 2015, soil pH increased significantly, with available concentrations of Zn, Pb, Cu and Cd decreasing by 13%, 81%, 77%, and 67%, respectively, and potential ecological risk indexes (*E_r_*) of the determined metals decreasing by more than 60%. Horizontally, total contents and percentages of available concentrations of Zn, Pb, Cu, and Cd decreased with the distance from the tailing heap in SD village, while pH values showed the reverse pattern. Vertically, Zn and Cd, Pb, and Cu showed similar vertical distribution patterns in the soil profiles. There was a slight downward migration for the determined metals in soil of M and H area and the mobility was in the order of Cd > Zn > Pb > Cu. It can be concluded that although concentrations and ecological risks of Cd, Cu, Pb, and Zn in soil decreased significantly, SD village is still a high risk area, and the priority pollutant is Cd.

## 1. Introduction

Metal pollution is covert, persistent, and irreversible [1]. This kind of pollution not only degrades the quality of the atmosphere, water bodies, soil, and food crops, but also threatens the health of animals and human beings [2,3]. For example, Pb and Cd are both non-essential elements to the human body, and long-term environmental exposure to Cd and Pb probably increases mortality rates of all cancer types (e.g., enteron tumours) [4]. Besides, exposure to high levels of Cu can cause mental diseases such as Alzheimer’s and Manganism [5]. The intake of excessive Zn can lead to a sideroblastic anemia [6].

Mineral resources is one of the basic sources for production and living, and the exploitation of mineral resources plays an important role in national economic development. However, it also induces many environmental problems. Large amounts of metals—such as Pb, Zn, Cd, and Cu—have been released into the environment due to mining activities, which have seriously contaminated water, soils, vegetables, and crops [7]. A recent survey showed that 33.4% of soils from the survey mining regions were contaminated by cadmium, lead, arsenic, and polycyclic aromatic hydrocarbons in China [8].

Pb and Zn resources are abundant in China, with reserves of 100 million tons being the second highest in the world [9]. The problems associated with excessive mining and waste disposal were severe in recent decades. In the past, tailings were directly disposed in the vicinity of the mine field as tailing dam [10,11], which is considered one of the worst environmental problems and leads to a serious hazard to ecosystems and human health [12,13]. A tailing dam of a lead/zinc mine in Chenzhou of China collapsed because of heavy rain in 1985, which significantly elevated the concentrations of Pb (452–1279 mg/kg), Zn (559–1165 mg/kg), Cd (3.98–12.19 mg/kg), and Cu (54.7–104.7 mg/kg) in topsoil [14]. After eight years, a survey in the same place showed that total contents of the metals varied slightly, which indicated that the leaching and plant extraction did not obviously decrease the metal levels in soils from this lead/zinc during the intervening eight years [15].

The tailing dam of Yangshuo (YS) Pb–Zn mine in Southern China collapsed in the 1970s due to a storm flood. Soil from SD village nearby the tailing heap was significantly contaminated. The mean concentrations of Zn, Pb, Cu, and Cd in topsoil in 1986 were 3936, 2007, 239, and 14 mg/kg, respectively, which were much higher than the national standard values of 200, 50, 50, and 0.3 mg/kg, respectively [16,17]. Some remedial measures such as alien earth, cultivation of non-food crops, and application of lime and fertilizers, have been taken to decrease metal levels in soil from the area since the collapse. In order to explore the pollution pattern after 40 years of remediation activities, and thus to assess the ecological risks, the soil profile samples were collected, and the total contents and BCR (Community Bureau of Reference, European Commission) sequential extraction fractions of Zn, Pb, Cu, and Cd were analyzed. The objectives of this paper are: (1) to investigate horizontal and vertical distribution patterns of the metals in soil; and (2) to compare soil concentrations and ecological risks of the determined metals between 1986 and 2015.

## 2. Materials and Methods

### 2.1. Study Area

**Figure 1:**
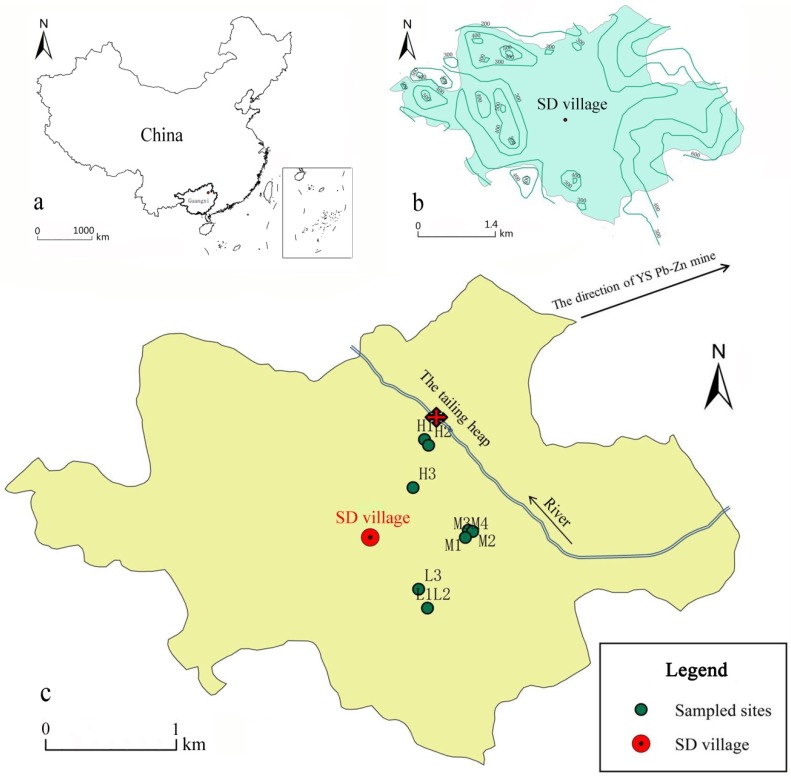
The study area (**a**), the topographic condition (**b**), and sampling sites (**c**).

SD village (110°33′ E, 24°58′ N) is located in the north-eastern suburb of Yangshuo, Guangxi Zhuang Autonomous Region, China. The average altitude is 150 m. Its average annual temperature and precipitation are 28.5 °C and 2000 mm. The local soil was developed from the sandy shale and limestone, which formed the hydromorphic paddy soil [18]. A river flows through this village and in the source region of this river, a YS Pb–Zn mine has been exploited since the 1950s [19]. The tailing dam collapsed in the 1970s due to a storm flood. The tailing sand thronged the river and swarmed into the farmland from a break point of the river channel. The tailing sand with the river piled up near the break point and became a tailing heap as shown in Figure 1. Later, the tailing heap and contaminated farmland was levelled off by the local people for crops or gardening. The remediation measurements taken in SD village included cementing the river channel, alien earth, cultivating non-food crops, and adding lime and fertilizers to polluted soils since 1975 to 1986, and cultivating non-food crops and adding lime and fertilizers to soil since 1986 to 2015. Compared with a large area of paddy field in 1986, citrus orchards became the main agricultural land in 2015, while there are only a few plots for paddy, corn, and vegetable fields in SD village now. 

### 2.2. Sample Collection

The tailing heap, which is located in the northeast of SD village, is surrounded by mountains in the north and east direction, and farm land is in the south and west direction. The soil samples were collected in the farm land based on the distance to the tailing heap. Ten topsoil samples were collected at different distances from the tailing heap (42, 75, 468, 756, 792, 803, 832, 1185, 1280, and 1296 m) in SD village in December 2015. Based on the distance between sampling sites and the tailing heap, 10 sampling sites were divided into three groups: H area (0–500 m), M area (500–1000 m), and L area (>1000 m), among which three belonged to H area (H1, H2, and H3) and L area (L1, L2, and L3), and four samples (M1, M2, M3, and M4) belonged to M area (Figure 1).

In each sampling site, soil samples at three different depths were collected, and a composite sample was made up of three subsamples. Soil samples were sealed in clean polyethylene bags, and were then air-dried, finely ground and passed through a 20-mesh sieve and a 100-mesh sieve in the laboratory. Total metal concentrations, BCRs, total organic carbon (TOC), and the pH values were determined for the sieved soils.

In order to compare the metal pollution level after the remediation activities, historical metal pollution data including total and available concentrations in 1986 were collected from Lin [17]. 

### 2.3. Analysis Methods

Adding 25 mL of cooled-down boiled ultrapure water to 10 g soil passed through the 20-mesh sieve in a small beaker (liquid:soil = 2.5:1), stirring for 1 min with a glass rod, sitting for half an hour at room temperature, soil pH was then measured by a pH meter with a glass electrode (INESA PHSJ-3F, INESA INSTRUMENT, Shanghai, China).

TOC were analyzed on Vario TOC Cube, Elementar High TOCII (Elementar Analysensysteme GmbH, Langenselbold, Germany). Fifteen mg of 100-mesh soil sample was placed in tin capsules and injected in a dry combustion chamber at 950 °C, 1000 mbar pressure. The NDIR infrared sensor (Elementar Analysensysteme GmbH, Langenselbold, Germany) quantified the evolved CO_2_ gas from each sample, and the instrument calculated TOC value (%) in the weight unit of the sample.

To determine the total contents of Pb, Zn, Cu, and Cd in the soil samples, a 10 mL mixture of HF–HNO_3_–HClO_4_ (5:4:1) was added to 0.05 g of the sample passed through the 100-mesh sieve in polytetrafluoroethylene tubes. These were kept overnight and then gently heated on the hot plate at 180 °C until a transparent extract was obtained. The digested extracts were transferred into glass tubes and diluted up to 20 mL volume using ultrapure water and kept at –4 °C for further analysis.

The BCR sequential extraction method divided metals into acid extractable fraction (exchangeable and bound to carbonates fraction), easily reducible fraction (bound to Fe/Mn oxides fraction), oxidizable fraction (bound to organic matter fraction), and residual fraction [20]. The modified procedures were as follows:

Step 1: Add 40 mL of 0.11 mol/L acetic acid to 1 g soil sample passed through the 100-mesh sieve in a 100-mL centrifuge tube. Extract for 16 h at 22 ± 5 °C using the horizontal shaker. Separate at 3000 r/min for 20 min by centrifugation, and decant the supernatant for analysis by ICP-MS. Add 20 mL ultrapure water to leftover, shake for 15 min at 3000 r/min, dump the supernatant, and leave behind the wet solid.

Step 2: Add 40 mL of 0.5 mol/L hydroxylamine hydrochloride solute to the residue from step 1 and shake for 16 h at 22 ± 5 °C. Centrifuge for 15 min at 3000 r/min by centrifugation, and decant the supernatant for analysis by ICP-MS. Repeat the washing steps in step 1.

Step 3: Add 10 mL of 8.8 mol/L hydrogen peroxide to the residue. Digest for 1 h at room temperature before digesting at 85 °C in a water bath until the volume reduces to 3 mL. Add 10 mL of hydrogen peroxide again and digest again at 85 °C until the volume is reduced to 1 mL. After the samples cool to room temperature, make up to 50 mL using 1 mol/L ammonium acetate adjusted to pH 2 by nitric acid. Shake for 16 h, centrifuge the extract and decant the supernatant for analysis. Wash the residue as described above in step 1.

Residual Step: Dry residual in a water bath before milling. Add 3, 2, 1, and 5 mL of HCl, HNO_3_, HClO_4_, and HF, respectively, to 0.1 g residue in polytetrafluoroethylene beakers, and heat until white smoke of perchloric acid disappears in a hot plate. Add 1 mL of 1:1 HCl and heat until salt dissolves. After cooling, sample digests were transferred into a 10 mL volumetric flask and brought to volume with ultrapure water before analysis by ICP-MS.

Available concentrations of metals in 1986 were collected from Lin [17], and available concentrations of metals in 2015 were the acid-extractable fraction concentrations in BCR sequential extraction method [21,22]. Zn and Pb concentrations in the soil samples were measured by inductive coupled plasma optical emission spectrometry (ICP-OES, ICP-OES 5300DV, PerkinElmer Instrument Co., Shelton, CT, USA), the measurement conditions included nebulizer gas flow (0.8 L/min), power (1300 w), observation distance (15 mm), plasma gas flow (15 L/min), and plasma observation direction (axial direction). Cu and Cd concentrations were measured by inductive coupled plasma mass spectrometry (ICP-MS, ELAN DRC-e, PerkinElmer Instrument Co., Shelton, CT, USA), the measurement conditions included nebulizer gas flow (0.83 L/min), ICP RF power (1100 w), auxiliary gas flow (1.2 L/min), plasma gas flow (15 L/min), and lens voltage (8.25 V). The standard reference materials of the soil sample (soil composition analysis standard substance GBW07410, Heilongjiang Environmental Monitoring Center, Harbin, China; soil for extractable trace elements GBW07437, National Research Center for Geoanalysis and National Institute of Metrology, Beijing, China) were digested and analyzed along with samples for quality control, and the metal recoveries ranged from 90.01% to 96.77%. Average values of three replicates of standards were taken for each determination. Quantification of metals was based on calibration curves of standard solutions of metals. These calibration curves were determined once every ten samples during the period of analysis. If the difference between values of standard solutions were beyond 10%, the calibration curves would be redone and samples would be re-determined. Metals’ total contents, sequential extraction concentrations, TOC, and pH of all the soil samples in 2015 can be found in Supplementary Materials (Table S1).

### 2.4. Ecological Risk Assessment

The potential ecological risk index (PERI) was invented by Hakanson [23] and has been widely used to assess the ecological risks of soil metals [24,25,26,27]. The formulas were defined as

(1) $$C_{r}=C/C_{0}$$

(2) $$E_{r}=T_{r}*C_{r}$$

(3) $$\mathrm{RI}=\sum_{i=1}^{n}E_{r}^{i}$$

where RI is integrated potential ecological risk index and represents the sensitivity of various biological communities to harmful elements and illustrates the potential ecological risk; *E_r_* is the potential ecological risk index of single element; *T_r_* is the ‘toxic-response’ factor for the given element (i.e., Cd = 30, Pb = Cu = 5, Zn = 1); *C_r_* is the pollution factor of the given element; *C* is the concentration of the given element in the topsoil; and *C*_0_ is the national standard of the given element [16].

### 2.5. Statistical Analysis

Data preparation was conducted in Microsoft Excel^®^ (Microsoft, Redmond, WA, USA). Statistical analyses including normal test, Student’s *t*-test, and correlation analysis were performed in SPSS 17.0 (IBM, Armonk, NY, USA).

## 3. Results

### 3.1. Concentrations of Metals

Table 1 shows that total contents of Pb, Zn, Cu, and Cd in 1986 were significantly higher than the standards for farmland in China [16]. In 2015, the mean concentrations of Zn, Pb, Cu, and Cd were reduced to 1302, 768, 82.6, and 4.82 mg/kg, respectively. They were still much higher than the standard values. However, the minimum concentrations of metals, except for Cd, were lower than the standard values. The concentrations of Zn, Pb, Cu, and Cd in topsoil from SD village decreased significantly from 1986 to 2015.

The topsoil pH in SD village has increased with time, with the range increased from 4.9–5.0 in 1986 to 4.98–7.45 in 2015, and the difference was significant (*p* = 0.001).

As shown in Table 2, the mean available concentrations of Zn, Pb, Cu, and Cd decreased by 13%, 81%, 77%, and 67%, respectively, since 1986, among which, only the decrease of available Cd concentrations was significant (*p* < 0.05).

### 3.2. Horizontal and Vertical Distribution (0–60 cm) of Metals in 2015

**Figure 2:**
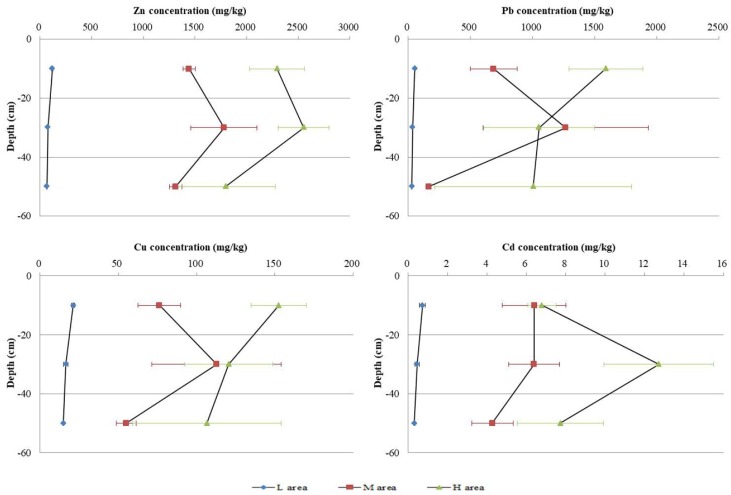
Concentrations of Zn, Pb, Cd, and Cu in different depths of L, M, and H areas in 2015.

The horizontal distributions of Zn, Pb, Cd, and Cu (Figure 2) in different depths all showed H area > M area > L area, except that of Pb in 20–40 cm, indicating that soil metal concentrations decreased with increasing distance from the tailing heap.

The vertical distributions of soil metals are shown in Figure 2. Zn and Cd, and Pb and Cu, show similar vertical distribution patterns in soil profiles. In the L area, metal concentrations decreased with soil depth, and there was no obvious difference among concentrations of the three layers. In M area, the maximum concentrations of these metals were all in the layer of 20–40 cm, and the minimum concentrations were in the 40–60 cm layer. In H area, the maximum concentrations of Zn and Cd still showed in the second layer, while those of Pb and Cu showed in the topsoil, and the metal concentration differences among different layers were bigger than those of the L area.

**Figure 3:**
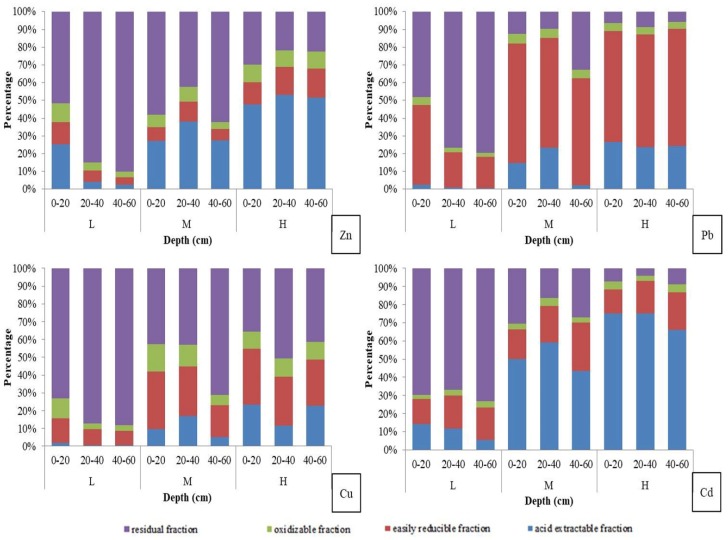
BCR (Community Bureau of Reference, European Commission) sequential extraction percentages of Zn, Pb, Cu, and Cd in different depths of L, M, and H area.

As highly toxic and the most bioavailable fraction [28], the acid-extractable fraction of Zn, Pb, Cu, and Cd in soil from the study area accounted for 2.40–53.25%, 0.68–26.51%, 0.55–23.32%, and 5.42–75.08% of their total contents, respectively. The acid-extractable fraction of Zn, Pb, Cu, and Cd was the highest in H area, followed by M area and L area. Under highly acidic or reducing conditions, an easily reducible fraction can be easily released into the environment and become available for uptake by living organisms [29,30,31]. The easily reducible fraction of Pb was 17.47–67.15%, which was higher than the other three metals and suggested that Pb could easily form stable complexes with Fe or Mn oxides. In oxidizable fraction, trace elements are bound to the organic matter and sulphides and they are released into the environment when the conditions become oxidative [22,29]. Oxidizable fractions accounted for 3.30–10.51%, 2.51–5.34%, 3.27–15.55%, and 2.15–4.54% of the total metal content of Zn, Pb, Cu, and Cd, respectively. Among the four BCR fractions of Zn, Pb, Cu, and Cd, oxidizable fractions accounted for the smallest proportion. Trace elements found in the residual fraction were strongly bound to the crystalline structures of the minerals present in the soil matrices, and studies have proven that these metals are not labile [29]. The proportion of this part in Zn, Pb, Cu, and Cd was 21.89–90.10%, 5.79–79.35%, 35.75–88.21%, and 4.03–73.17%, respectively. As seen in Figure 3, proportions of residual fraction in Zn, Pb, Cu, and Cd decreased from L area to H area, which was opposite to the variation tendency of acid extractable fraction.

The vertical distribution of acid extractable fraction was similar to that of the total content of Zn, Pb, Cu, and Cd (Figure 2 and Figure 3). In L area, percentages of acid extractable fraction decreased with soil depth, and the maximum acid extractable fraction percentages of Zn, Pb, Cu, and Cd were found in the layer of 20–40 cm in M area. In H area, the maximum acid extractable fraction percentages of Zn and Cd still showed in the second layer, while for Pb and Cu, the maximum acid extractable fraction percentages showed in the topsoil. The vertical distribution patterns of acid extractable fraction were similar for Zn and Cd, and for Pb and Cu.

Furthermore, Spearman correlation coefficients showed that there was significantly negative correlation between soil pH and acid-extractable fraction concentrations of Zn, Pb, Cu, and Cd (r = −0.603 **, −0.570 **, −0.583 **, and −0.596 **, and “**” means correlation is significant at the 0.01 level). Soil TOC also affects the speciation of soil metals. The range of soil TOC was 0.34–1.85% in 0–60 cm soil in 2015, and the order was M area > L area > H area in 0–20 cm. There was positive correlation between soil TOC and acid-extractable fraction concentrations of Pb and Cu (r = 0.396 * and 0.403 *, and “*” means correlation is significant at the 0.05 level), and positive correlation between soil TOC and oxidizable fraction concentrations of Zn, Pb, and Cu (r = 0.417 *, 0.440 *, and 0.570 **).

### 3.3. Potential Ecological Risk Assessment of Zn, Pb, Cu, and Cd in Topsoil between 1986 and 2015

Table 3 shows that the mean *E_r_* values for Zn and Cu were less than 40, the mean *E_r_* for Pb was 201, while mean *E_r_* for Cd was 1390 in 1986. In 2015, *E_r_* of the determined metals decreased by more than 60% compared with the 1986 values. The RI (integrated potential ecological risk index) of metals also declined from 1634 to 557. However, the mean *E_r_* for Cd in 2015 was still higher than 320, so the potential ecological risk remained at a dangerous level and the priority pollutant was Cd.

For the topsoil in 1986, *E_r_* values indicated that Zn and Cu had low potential ecological risks, Pb showed very high-potential ecological risk and Cd posed a dangerous risk. In 2015, the ecological risks for Zn, Cu, and Cd have not changed, while for Pb, the ecological risks degraded to moderate risk levels. Comparing the *E_r_* and RI values between 1986 and 2015, the ecological risks of Zn, Pb, Cu, and Cd all declined. Still, the potential ecological risk was at a dangerous level and the major contributor was Cd.

## 4. Discussion

Due to the remediation activities, total contents and available concentrations of Zn, Pb, Cu, and Cd in the topsoil of SD village all decreased since 1986, and related ecological risks decreased correspondingly. According to previous research, adding lime and manure to acid and polluted paddy soils increased soil pH and decreased available Cd concentrations, and alien earth and planting non-food crops obviously decreased paddy soil Cd concentration in SD village [17]. Through the neutralization of the acid mine discharge, lime and fertilizers have increased soil pH and decreased the metals availability in soil [17,32]. With these remediation measures, from 1986 to 2015, the pH value increased from 4.9 to 6.2, total contents of Zn, Pb, Cu, and Cd decreased by 66.9%, 61.7%, 65.4%, and 65.3%, respectively, and the available concentrations decreased by 13%, 81%, 77%, and 67%, respectively. Therefore, the remediation through neutralization was effective in SD village after tailing collapse.

In general, soil metal concentrations decreased with increasing distance from the polluting source [33]. In SD village, the total contents of Zn, Pb, Cu, and Cd decreased with the distance from the tailing heap. The altitude in SD village is L area > H area > M area. Therefore, after being incorporated into the soil, metals from the tailing heap might diffuse from H area to M area through soil runoff, but might not from M area to L area, leading to decreased metal concentrations with increasing distance from the tailing heap and a similar metal vertical distribution pattern between H and M area. Meanwhile, percentages of acid-extractable fraction decrease with the distance, which might be due to pH values increasing with distance in this study. Soil pH can affect metal physiochemical parameters, and acidification causes the dissolution of metal compounds, increasing the concentration of metals in soil solution [34]. Most sequential extraction studies report that metal mobility (acid-extractable fraction concentrations/available concentrations) is dependent on pH and the presence of secondary minerals [35,36]. In this study, a significant negative correlation was found between acid-extractable fraction concentrations of Zn, Pb, Cu, and Cd and soil pH.

Zn and Cd, and Pb and Cu, showed similar vertical distribution patterns of total contents and available concentrations in soil profiles in SD village. In L area, total contents and available concentrations of Zn, Cd, Pb, and Cu were the highest in topsoil, with a slow decrease with depth. However, in M area, the highest total contents and available concentrations of the four metals were found at 20–40 cm, indicating the metals have been migrating from the topsoil and enriched at a depth of 20–40 cm. For H area, Zn and Cd have migrated and enriched at 20–40 cm, while Pb and Cu showed the highest total contents and available concentrations in the topsoil. This downward migration pattern has also been found in many studies [37,38,39]. Zhang et al. [40] indicated that the metal accumulation in the surface soil would migrate to the bottom of the soil under long-term accumulation, weathering, and leaching, and showed a cumulative effect. The intensity and frequency of agricultural activities in M area were stronger than in H area of SD village. Deep ploughing and soaking provided preferable weathering and leaching conditions and accelerated metals’ mobility, which might relate to the accumulation of Zn, Cd, Pb, and Cu at 20–40 cm in M area and the accumulation of Zn and Cd at 20–40 cm in H area [32,41].

Several studies have addressed the metals’ mobility as a measure of the amount present in the labile phase and therefore available to living organisms [42,43,44]. The BCR sequential extraction method is a common method, in which the percentage of acid-extractable fraction is always used to measure the metals’ mobility [45,46,47]. For SD village, the mean acid-extractable fraction percentages of Zn, Pb, Cu, and Cd were 30.84%, 13.23%, 10.27%, and 44.48%, respectively, so the mobility was in the order of Cd > Zn > Pb > Cu. Compared with Pb and Cu, Cd and Zn have stronger downward mobility and thus reach a greater depth in soil profiles, which is consistent with some research results [32,38]. Besides, soil TOC had positive correlation with acid-extractable and oxidizable fraction concentrations of Pb and Cu, which may contribute to the weaker downward mobility of Pb and Cu. Liu et al. [48] found that the organic matter content was positively correlated with exchangeable and organic combined Pb, which implied the increase of organic matter would transform carbonate combined Pb to organic combined Pb and thus decreased the mobility of Pb.

Land use type was also suggested as an explanatory variable for the spatial variation of metal concentrations [34]. Some studies found that traffic and intensive human activities contributed to Pb contamination and elevated soil Zn was associated with industrial and agricultural land uses [49,50]. In this study, a significant difference for Cd concentrations was found between paddy and vegetable field (*p* = 0.098), but not for the other three metals. This might be attributable to crop rotation and mixed cultivation in SD village.

Reclamation of agricultural soil contaminated by the Pb–Zn mine is a lengthy undertaking. This study reveals that, with decades of remediation activities, the metal concentrations decreased significantly, but the pollution level is still well above the national standards, and the pollutants are migrating from the top to deeper levels. More remediation measures should be taken in SD village.

## 5. Conclusions

Though still exceeding the national standards, the total contents and the available concentrations of Zn, Pb, Cu, and Cd decreased significantly in SD village from 1986 to 2015, and correspondingly, the potential ecological risk decreased by more than 60%. A slight metal migration from topsoil to 20–40 cm of H and M areas was identified, of which, Zn and Cd, and Pb and Cu showed identical downward migration patterns with the mobility in the order of Cd > Zn > Pb > Cu. The soil in SD village was still at high or even dangerous risk level, and the priority pollutant was Cd.

## Figures and Tables

**Table 1:** Total contents of topsoil (0–20 cm) metals in 1986 and 2015 (mg/kg).

Metals and Soil pH	1986 [17] ^1^		2015 ^2^		*p*	National Standards [16]
	Mean	Range	Mean	Range		
Zn	3935.60	1703.20–7216.60	1301.79	107.68–2822.98	0.004	≤200
Pb	2006.60	886.20–3579.20	768.41	46.54–2024.00	0.015	≤50
Cu	238.50	167.80–420.40	82.60	19.35–171.80	0.002	≤50
Cd	13.90	11.80–18.50	4.82	0.47–11.20	0.000	≤0.3
soil pH	4.90	4.90–5.00	6.24	4.98–7.45	0.001	<6.5

^1^*n* = 5; ^2^ *n* = 10.

**Table 2:** Available concentrations of topsoil (0–20 cm) metals in 1986 and 2015 (mg/kg).

Metals	1986 [17]		2015		*p*
	Mean	Range	Mean	Range	
Zn	628.60	418.00–1192.00	547.23	15.33–1601.17	0.757
Pb	989.50	453.00–2617.00	188.60	0.76–635.49	0.122
Cu	63.50	31.70–172.00	14.38	0.550–52.99	0.146
Cd	7.80	5.90–9.40	2.60	0.18–5.68	0.000

**Table 3:** PERI (potential ecological risk index) of Zn, Pb, Cu, and Cd in topsoil (0–20 cm) of SD village between 1986 and 2015.

Index	$E_{r}$-1986				$E_{r}$-2015				RI-1986	RI-2015
	Zn	Pb	Cu	Cd	Zn	Pb	Cu	Cd		
Min	9	89	17	1180	1	5	2	47		
Max	36	358	42	1850	14	202	17	1120		
Mean	20	201	24	1390	6	78	8	464	1634	557
PERI ^3^ [23]	$E_{r}$^1^ < 40; RI ^2^ < 150				Low potential ecological risk					
	40 ≤ $E_{r}$ < 80; 150 ≤ RI < 300				Moderate potential ecological risk					
	80 ≤ $E_{r}$ < 160; 300 ≤ RI < 600				Considerable potential ecological risk					
	160 ≤ $E_{r}$ < 320; RI ≥ 600				Very high potential ecological risk					
	$E_{r}$ ≥ 320				Dangerous					

^1^$E_{r}$ is the potential ecological risk index of single element; ^2^ RI is integrated potential ecological risk index; ^3^ PERI is the potential ecological risk index.

## Supplementary Materials

The following are available online at http://www.mdpi.com/1660-4601/15/5/940/s1, Table S1: Metals’ total contents, BCR sequential extraction concentrations, TOC and pH of all the soil samples in 2015.

## References

[1] Wang Q.R., Dong Y., Cui Y., Liu X. (2001). Instances of soil and crop heavy metal contamination in China. Soil Sediment Contam..

[2] Dong J., Yang Q.W., Sun L.N., Zeng Q., Liu S.J., Pan J., Liu X.L. (2011). Assessing the concentration and potential dietary risk of heavy metals in vegetables at a Pb/Zn mine site, China. Environ. Earth Sci..

[3] Nabulo G., Young S.D., Black C.R. (2010). Assessing risk to human health from tropical leafy vegetables grown on contaminated urban soils. Sci. Total Environ..

[4] Wang M., Song H., Chen W.Q., Lu C.Y., Hu Q.S., Ren Z.F., Yang Y., Xu Y.J., Zhong A.M., Ling W.H. (2011). Cancer mortality in a Chinese population surrounding a multi-metal sulphide mine in Guangdong province: An ecologic study. BMC Public Health.

[5] Dieter H.H., Bayer T.A., Multhaup G. (2005). Environmental copper and manganese in the pathophysiology of neurologic diseases (Alzheimer’s disease and Manganism). Acta Hydrochim. Hydrobiol..

[6] Muhammad S., Shah M.T., Khan S. (2011). Health risk assessment of heavy metals and their source apportionment in drinking water of Kohistan region, northern Pakistan. Microchem. J..

[7] Zhang X.W., Yang L.S., Li Y.H., Li H.R., Wang W.Y., Ye B.X. (2012). Impacts of lead/zinc mining and smelting on the environment and human health in China. Environ. Monit. Assess..

[8] Ministry of Environmental Protection of the People’s Republic of China, Ministry of Land and Resources of the People’s Republic of China (CMEP and CMLR) National Soil Pollution Survey Communique. http://www.zhb.gov.cn/gkml/hbb/qt/201404/t20140417_270670.htm.

[9] Zhang C.Q., Liu H., Wang D.H., Chen Y.C., Rui Z.Y., Lou D.B., Wu Y., Jia F.D., Chen Z.H., Meng X.Y. (2015). A preliminary review on the metallogeny of Pb-Zn deposits in China. Acta Geol. Sin.-Engl..

[10] Pascaud G., Boussen S., Soubrand M., Joussein E., Fondaneche P., Abdeljaouad S., Bril H. (2015). Particulate transport and risk assessment of Cd, Pb and Zn in a Wadi contaminated by runoff from mining wastes in a carbonated semi-arid context. J. Geochem. Explor..

[11] Johnson A.W., Gutiérrez M., Gouzie D., McAlily L.R. (2016). State of remediation and metal toxicity in the tri-state mining district, USA. Chemosphere.

[12] Fields S. (2003). The Earth’s open wounds: Abandoned and orphaned mines. Environ. Health Perspect..

[13] Hudson-Edwards K.A., Jamieson H.E., Lottermoser B.G. (2011). Mine wastes: Past, present, future. Elements.

[14] Zeng Q.R., Zhou X.H., Tie B.Q., Yang R.B. (1997). Study on characteristics of heavy metal pollution and its controlling measures in lead–zinc mine area. Rural Eco-Environ..

[15] Liu H.Y., Probst A., Liao B.H. (2005). Metal contamination of soils and crops affected by the Chenzhou lead/zinc mine spill (Hunan, China). Sci. Total Environ..

[16] Ministry of Environmental Protection of the People’s Republic of China (CMEP) Farmland Environmental Quality Evaluation Standards for Edible Agricultural Products (HJ/T 332-2006). http://kjs.mep.gov.cn/hjbhbz/bzwb/stzl/200611/t20061122_96418.htm.

[17] Lin B.Y. (1997). Study of cadmium pollution in soil-crop system of lead-zinc deposit in Guangxi province, China. Chin. J. Soil Sci..

[18] Jin Z.J., Li Z.Y., Li Q., Hu Q.J., Yang R.M., Tang H.F., Li M., Huang B.F., Zhang J.Y., Li G.W. (2015). Canonical correspondence analysis of soil heavy metal pollution, microflora and enzyme activities in the Pb–Zn mine tailing dam collapse area of Sidi village, SW China. Environ. Earth Sci..

[19] Qin C.K., Li Y., Wei S., Huang G.Y. (2005). Analysis on present environmental situation and treatment model of tailing waste water in Yangshuo lead-zinc deposit. Min. Resour. Geol..

[20] Rauret G., Lopez-Sanchez J.F., Sahuquillo A., Rubio R., Davidson C., Ure A., Quevauviller P. (1999). Improvement of the BCR three step sequential extraction procedure prior to the certification of new sediment and soil reference materials. J. Environ. Monit..

[21] He Z.L.L., Yang X.E., Stoffella P.J. (2005). Trace elements in agroecosystems and impacts on the environment. J. Trace Elem. Med. Biol..

[22] Bakircioglu D., Kurtulus Y.B., Ibar H. (2011). Investigation of major and trace elements in agricultural soils by BCR sequential extraction method and its transfer to wheat plants. Environ. Monit. Assess..

[23] Hakanson L. (1980). An ecological risk index for aquatic pollution control: A sedimentological approach. Water Res..

[24] Yuan G.L., Sun T.H., Han P., Li J., Lang X.X. (2014). Source identification and ecological risk assessment of heavy metals in topsoil using environmental geochemical mapping: Typical urban renewal area in Beijing, China. J. Geochem. Explor..

[25] Zhao W.T., Ding L., Gu X.W., Luo J., Liu Y.L., Guo L., Shi Y., Huang T., Cheng S.G. (2015). Levels and ecological risk assessment of metals in soils from a typical e-waste recycling region in southeast China. Ecotoxicology.

[26] Pan L.B., Ma J., Hu Y., Su B.Y., Fang G.L., Wang Y., Wang Z.S., Wang L., Xiang B. (2016). Assessments of levels, potential ecological risk, and human health risk of heavy metals in the soils from a typical county in Shanxi Province, China. Environ. Sci. Pollut. Res..

[27] Tepanosyan G., Sahakyan L., Belyaeva O., Saghatelyan A. (2016). Origin identification and potential ecological risk assessment of potentially toxic inorganic elements in the topsoil of the city of Yerevan, Armenia. J. Geochem. Explor..

[28] Wang S.F., Jia Y.F., Wang S.Y., Wang X., Wang H., Zhao Z.X., Liu B.Z. (2010). Fractionation of heavy metals in shallow marine sediments from Jinzhou Bay, China. J. Environ. Sci..

[29] Nemati K., Bakar N.K.A., Abas M.R.B., Sobhanzadeh E., Low K.H. (2011). Comparison of unmodified and modified BCR sequential extraction schemes for the fractionation of heavy metals in shrimp aquaculture sludge from Selangor, Malaysia. Environ. Monit. Assess..

[30] Fathollahzadeh H., Kaczala F., Bhatnagar A., Hogland W. (2014). Speciation of metals in contaminated sediments from Oskarshamn Harbor, Oskarshamn, Sweden. Environ. Sci. Pollut. Res..

[31] Ma X.L., Zuo H., Tian M.J., Zhang L.Y., Meng J., Zhou X.N., Min N., Chang X.Y., Liu Y. (2016). Assessment of heavy metals contamination in sediments from three adjacent regions of the Yellow River using metal chemical fractions and multivariate analysis techniques. Chemosphere.

[32] Gutiérrez M., Mickus K., Camacho L.M. (2016). Abandoned Pb-Zn mining wastes and their mobility as proxy to toxicity: A review. Sci. Total Environ..

[33] Liang Y.Y., Yi X.Y., Dang Z., Wang Q., Luo H.M., Tang J. (2017). Heavy metal contamination and health risk assessment in the vicinity of a tailing pond in Guangdong, China. Int. J. Environ. Res. Public Health.

[34] Hou D.Y., O’Connor D., Nathanail P., Tian L., Ma Y. (2017). Integrated GIS and multivariate statistical analysis for regional scale assessment of heavy metal soil contamination: A critical review. Environ. Pollut..

[35] Byrne P., Reid I., Wood P.J. (2010). Sediment geochemistry of streams draining abandoned lead/zinc mines in central Wales: The Afon Twymyn. J. Soils Sediments.

[36] Ciszewski D., Aleksander-Kwaterczak U., Pociecha A., Szarek-Gwiazda E., Waloszek A., Wilk-Woźniak E. (2013). Small effects of a large sediment contamination with heavy metals on aquatic organisms in the vicinity of an abandoned lead and zinc mine. Environ. Monit. Assess..

[37] Liénard A., Colinet G. (2016). Assessment of vertical contamination of Cd, Pb and Zn in soils around a former ore smelter in Wallonia, Belgium. Environ. Earth. Sci..

[38] Qi J.Y., Zhang H.L., Li X.P., Lu J., Zhang G.S. (2016). Concentrations, spatial distribution, and risk assessment of soil heavy metals in a Zn-Pb mine district in southern China. Environ. Monit. Assess..

[39] Qu L., Xie Y.Y., Lu G.N., Yang C.F., Zhou J.N., Yi X.Y., Dang Z. (2017). Distribution, fractionation, and contamination assessment of heavy metals in paddy soil related to acid mine drainage. Paddy Water Environ..

[40] Zhang W.T., You M., Hu Y.H. (2016). The distribution and accumulation characteristics of heavy metals in soil and plant from Huainan coalfield, China. Environ. Progress Sustain. Energy.

[41] Ruan X.L., Zhang G.L., Zhao Y.G., Yuan D.G., Wu Y.J. (2006). Distribution and migration of heavy metals in soil profiles by high-resolution sampling. Environ. Sci..

[42] Schaider L.A., Senn D.B., Brabander D.J., McCarthy K.D., Shine J.P. (2007). Characterization of zinc, lead and cadmium in mine waste: Implications for transport, exposure, and bioavailability. Environ. Sci. Technol..

[43] Kapusta P., Szarek-Lukaszewska G., Stefanowicz A.M. (2011). Direct and indirect effects of metal contamination on soil biota in a Zn-Pb post-mining and smelting area (S Poland). Environ. Pollut..

[44] Galende M.A., Becerri J.M., Barrutia O., Artetxe U., Garbisu C., Hernández A. (2014). Field assessment of the effectiveness of organic amendments for aided phytostabilization of a Pb-Zn contaminated mine soil. J. Geochem. Explor..

[45] Anju M., Banerjee D.K. (2011). Associations of cadmium, zinc, and lead in soils from a lead and zinc mining area as studied by single and sequential extractions. Environ. Monit. Assess..

[46] Kerolli-Mustafa M., Fajković H., Rončević S., Ćurković L. (2015). Assessment of metal risks from different depths of jarosite tailing waste of Trepça zinc industry, Kosovo, based on BCR procedure. J. Geochem. Explor..

[47] Li P.Z., Lin C.Y., Cheng H.G., Duan X.L., Lei K. (2015). Contamination and health risks of soil heavy metals around a lead/zinc smelter in southwestern China. Ecotoxicol. Environ. Saf..

[48] Liu X., Liu S.Q., Wang S.A. (2003). Distribution of Cadmium and Lead forms and its affecting factors in soils of Hebei province. Acta Pedol. Sin..

[49] Davis H.T., Aelion C.M., McDermott S., Lawson A.B. (2009). Identifying natural and anthropogenic sources of metals in urban and rural soils using GIS-based data, PCA, and spatial interpolation. Environ. Pollut..

[50] Kheir R.B., Greve M.H., Abdallah C., Dalgaard T. (2010). Spatial soil zinc content distribution from terrain parameters: A GIS-based decision-tree model in Lebanon. Environ. Pollut..

