# Supplementary material for: Temporal Variation and Ecological Risk Assessment of Metals in Soil Nearby a Pb–Zn Mine in Southern China
Source: Int J Environ Res Public Health. 2018 May 9;15(5):940. doi: 10.3390/ijerph15050940 (PMC5981979; doi:10.3390/ijerph15050940)
Supplement: Supplementary file 1 [file ijerph-15-00940-s001.pdf]

Table S1. Metals total contents, BCR sequential extraction concentrations, TOC and pH of all the soil samples in 2015

Sampling area	Soil sample	soil layer (cm)	TOC (%)	pH	Cd (mg/kg)					Pb (mg/kg)					Cu (mg/kg)					Zn (mg/kg)				
					**Acid extractable fraction**	**Easily reducible fraction**	**Oxidizable fraction**	**Residual fraction**	**Total content**	**Acid extractable fraction**	**Easily reducible fraction**	**Oxidizable fraction**	**Residual fraction**	**Total content**	**Acid extractable fraction**	**Easily reducible fraction**	**Oxidizable fraction**	**Residual fraction**	**Total content**	**Acid extractable fraction**	**Easily reducible fraction**	**Oxidizable fraction**	**Residual fraction**	**Total content**
H area	H1	0-20	0.94	5.22	3.34	0.72	0.23	0.48	5.36	635.49	1557.03	105.72	114.58	2024.00	52.99	58.44	19.89	52.52	171.80	1059.80	270.48	250.56	533.60	2021.83
		20-40	0.48	6.03	4.90	0.64	0.23	0.52	8.20	634.67	1050.67	59.58	161.99	1929.00	25.07	43.01	14.17	87.90	172.40	1827.29	345.18	168.40	382.88	2257.27
		40-60	0.60	6.25	8.72	1.22	0.51	1.32	11.89	748.11	1772.83	94.33	78.72	2592.00	75.81	65.01	28.13	52.23	201.60	1305.90	453.48	284.25	532.94	2720.52
	H2	0-20	0.87	6.11	5.60	1.00	0.37	0.56	7.69	562.30	1145.45	89.88	124.42	1727.00	36.62	54.74	12.98	75.13	168.60	1601.17	407.65	254.45	813.13	2822.98
		20-40	0.93	6.37	12.07	3.39	0.52	0.55	17.80	73.28	660.88	46.27	60.37	766.30	11.99	38.92	16.33	55.39	113.20	1222.00	596.58	321.17	580.59	3038.80
		40-60	0.64	6.43	3.88	2.00	0.23	0.37	6.84	7.08	135.31	12.92	70.04	194.30	2.54	12.02	2.61	51.70	56.52	1068.48	260.97	131.40	264.98	1567.30
	H3	0-20	1.49	5.87	5.68	0.87	0.25	0.38	7.34	238.25	682.67	58.24	102.34	1024.00	23.64	39.13	13.57	45.94	117.20	979.97	267.86	244.91	927.56	2040.36
		20-40	1.22	6.24	9.51	2.28	0.31	0.35	12.19	62.03	362.92	30.74	59.58	463.30	7.58	24.30	8.20	52.53	76.03	1446.21	391.30	276.60	884.72	2361.91
		40-60	0.84	6.55	2.10	1.41	0.18	0.30	4.46	12.44	189.18	16.54	34.90	236.30	3.18	14.72	4.33	43.89	61.73	433.15	169.07	102.60	421.64	1107.32
M area	M1	0-20	0.99	5.46	1.96	0.56	0.13	4.75	11.20	23.76	157.23	16.81	93.45	252.40	3.41	10.39	4.40	30.35	54.00	259.81	63.25	58.83	1067.71	1284.99
		20-40	0.45	6.02	1.79	1.08	0.12	2.11	3.57	3.71	72.23	6.89	41.56	119.80	2.51	9.45	1.92	34.67	43.92	311.15	98.06	47.95	1277.92	1077.89
		40-60	0.54	6.12	0.95	0.74	0.10	1.41	3.22	3.23	102.39	8.71	79.39	172.80	2.71	9.43	3.80	50.34	61.33	168.10	63.89	42.60	1021.05	1254.47
	M2	0-20	1.29	4.98	2.78	0.66	0.17	0.90	4.15	261.12	869.19	59.57	77.88	1163.00	13.42	41.90	19.49	32.89	115.80	526.39	110.29	116.69	1043.12	1426.69
		20-40	0.70	5.99	6.90	2.52	0.33	0.38	9.82	11.61	142.88	11.91	32.10	188.60	2.99	12.01	3.04	31.93	44.83	1040.99	240.14	122.93	451.50	1396.17
		40-60	0.54	6.23	2.60	1.45	0.13	0.79	5.33	5.11	106.72	8.84	34.50	159.10	3.24	11.95	2.77	33.85	48.87	714.83	149.07	79.60	973.68	1372.19
	M3	0-20	1.39	6.78	3.00	0.83	0.21	0.35	4.90	113.86	495.60	49.21	108.43	734.00	7.04	21.15	11.08	28.54	69.44	349.85	124.04	132.44	994.61	1532.42
		20-40	1.03	6.98	3.33	0.79	0.40	0.61	6.28	802.51	1812.29	154.79	229.95	2797.00	45.73	58.76	29.26	72.61	210.90	1006.25	313.18	264.68	645.29	2351.01

	M4	0-20	1.85	6.96	2.74	1.33	0.20	0.36	5.34	46.08	485.05	34.09	97.53	596.90	4.65	21.26	10.83	33.82	65.51	590.29	181.26	146.99	550.17	1529.15
		20-40	1.17	7.10	3.38	0.84	0.34	1.15	5.92	461.64	1356.91	111.72	218.60	1964.00	26.42	49.52	21.09	59.69	151.70	915.48	303.83	281.79	1264.15	2299.78
L area	L1	0-20	1.26	6.95	0.25	0.27	0.04	1.63	0.70	3.06	30.80	3.09	32.19	55.92	0.55	4.36	3.07	24.65	19.35	16.80	11.68	12.25	101.05	114.56
		20-40	0.61	7.83	0.07	0.10	0.01	0.22	0.39	0.24	13.41	1.10	17.44	35.61	0.23	3.01	1.28	19.00	15.20	3.47	6.83	5.39	97.57	83.91
		40-60	0.36	7.84	0.03	0.08	0.02	0.32	0.34	0.64	9.12	1.02	16.65	27.00	0.20	2.85	1.22	18.56	15.09	2.85	4.74	3.31	94.81	70.40
	L2	0-20	1.55	6.65	0.48	0.45	0.06	2.49	1.00	1.28	35.03	3.32	47.06	60.35	0.80	5.26	5.44	24.67	23.23	72.96	29.91	20.81	54.58	137.23
		20-40	0.53	7.21	0.09	0.13	0.02	0.65	0.67	1.08	13.03	1.77	98.34	34.10	0.26	2.67	0.69	35.52	14.96	7.59	8.38	4.93	82.28	73.01
	L3	0-20	1.15	7.45	0.18	0.17	0.03	0.31	0.47	0.76	23.71	2.37	17.17	46.54	0.70	5.09	3.27	28.66	21.09	15.33	10.93	10.75	59.97	107.69
		20-40	0.47	8.06	0.02	0.05	0.01	0.17	0.30	0.19	7.75	1.65	15.97	34.86	0.12	2.39	1.02	25.22	19.23	1.36	2.96	3.01	69.09	73.56
		40-60	0.34	8.08	0.02	0.08	0.01	0.33	0.29	0.03	8.02	1.45	61.20	30.70	0.10	1.47	0.57	29.42	14.69	1.19	2.33	2.24	56.78	63.53
